# The ^13^C hyperpolarized pyruvate generated by ParaHydrogen detects the response of the heart to altered metabolism in real time

**DOI:** 10.1038/s41598-018-26583-2

**Published:** 2018-05-30

**Authors:** Eleonora Cavallari, Carla Carrera, Matteo Sorge, Gisèle Bonne, Antoine Muchir, Silvio Aime, Francesca Reineri

**Affiliations:** 1 Department of Molecular Biotechnologies and Health Sciences, University of Torino, Torino, Italy; 2 Sorbonne Université, Inserm UMRS974, Center of Research in Myology, Institut de Myologie, G.H. Pitie-Salpetriere, Paris, France

## Abstract

Many imaging methods have been proposed to act as surrogate markers of organ damage, yet for many candidates the essential biomarkers characteristics of the injured organ have not yet been described. Hyperpolarized [1-^13^C]pyruvate allows real time monitoring of metabolism *in vivo*. ParaHydrogen Induced Polarization (PHIP) is a portable, cost effective technique able to generate ^13^C MR hyperpolarized molecules within seconds. The introduction of the Side Arm Hydrogenation (SAH) strategy offered a way to widen the field of PHIP generated systems and to make this approach competitive with the currently applied dissolution-DNP (Dynamic Nuclear Polarization) method. Herein, we describe the first *in vivo* metabolic imaging study using the PHIP-SAH hyperpolarized [1-^13^C]pyruvate. *In vivo* maps of pyruvate and of its metabolic product lactate have been acquired on a 1 T MRI scanner. By comparing pyruvate/lactate ^13^C label exchange rate in a mouse model of dilated cardiomyopathy, it has been found that the metabolic dysfunction occurring in the cardiac muscle of the diseased mice can be detected well before the disease can be assessed by echocardiographic investigations.

## Introduction

^13^C Magnetic Resonance Spectroscopy (^13^C-MRS) is a noninvasive technique that allows *in vivo* detection and quantitation of metabolites^1,2^, but its practical application has been hampered by the intrinsic low sensitivity of MR based methods. The introduction of hyperpolarization techniques, that enhance the ^13^C-MR signals^3–5^, allowed the *in vivo* detection of ^13^C-MR signatures of endogenous molecules and the real time visualization of their by-products, within few tens of seconds from their injection^6–10^.

[1-^13^C]Pyruvate is at a central crossroad of cellular metabolism as it leads to energy production as well as to the formation of lactate, alanine and CO_2_. This has allowed for the *in vivo* study in animals of ^13^C metabolism with rapid scan times, good signal-to-noise ratio (SNR), and no background signal^11^. In particular, hyperpolarized [1-^13^C]pyruvate has the potential to observe cellular bioenergetics, such as glycolysis^12–14^ and the citric acid cycle^15,16^. Its conversion to [1-^13^C]lactate and [1-^13^C]alanine has been observed extensively *in vivo* in animal models. Moreover, clinical trials are currently carried out for validating the use of HP [1-^13^C]pyruvate in prostate cancer diagnosis^17^ and the first ^13^C metabolic MRI study of the human heart has been reported recently^18^.

The widespread application of this powerful diagnostic tool is limited to few laboratories worldwide, due to the high cost of the d-DNP (dissolution-Dynamic Nuclear Polarization) technique. ParaHydrogen Induced Polarization (PHIP)^19,20^ is a method for the generation of HP molecules^5,21,22^ that is based on the catalytic hydrogenation of substrates using hydrogen enriched in the para-spin isomer (i.e. *para*hydrogenation). A few prototypes for parahydrogen hyperpolarization have been reported^23–25^ and, albeit a hyperpolarizer is not commercially available, the costs can be estimated to be one-two orders of magnitude lower than a d-DNP.

The relatively low speed of the hyperpolarization process, that varies from 0.5 to 3 hours depending on the substrate of choice, is another major challenge of the d-DNP technique. ParaHydrogen hyperpolarization does not suffer from this limitation because, thanks to the rapid catalysis, hyperpolarized substrates can be produced in seconds while the entire polarization process takes few minutes.

*In vivo* molecular imaging studies using parahydrogen hyperpolarized substances have already been reported^23,26^ and a few metabolites have been hyperpolarized by means of PHIP^27–29^. Among the parahydrogen hyperpolarized substrates that have been designed for biomedical applications, hydroxyethylpropionate (HEP), succinate (SUC),) and phospholactate (PLac) have also been used *in vivo*. HEP has been used for catheter tracking and angiography, it does not undergo fast metabolic processes and its toxicity has to be investigated. Succinate is a metabolite and takes part to the citric acid cycle, where it is converted into fumarate. ^13^C-MR images have been obtained following to *in vivo* injection of 13C hyperpolarized SUC. Images have been also acquired following to injection of hyperpolarized Phospholactate, showing 13C-enhancement in the heart, kydneys and blood^23^.

Hyperpolarization of [1-^13^C]pyruvate was precluded to parahydrogen-based methods because of the problems related to the availability of unsaturated precursors of the target hyperpolarized bio-molecules.

In this perspective, PHIP-SAH (PHIP by means of Side Arm Hydrogenation) represented a breakthrough because parahydrogen is not incorporated in the end product. In the SAH approach, the molecule of interest is functionalized with an unsaturated moiety (the “side arm”) that, after parahydrogenation and hyperpolarization of the ^13^C target signal, is chemically removed. This strategy allows to hyperpolarize pyruvate, and other metabolites^30^, that, previously, could be obtained only by d-DNP^31^.

Alterations in myocardial energy metabolism have been thought to contribute to the development of heart failure. A non-invasive *in vivo* imaging of metabolic processes represents an invaluable diagnostic tool to quantify this shift in energy metabolism in the failing heart. In this work, HP [1-^13^C]pyruvate has been used to assess heart metabolism in normal and genetically modified *Lmna*^H222P/H222P^ mice that develop dilated cardiomyopathy^32^. Dilated cardiomyopathy is characterized by left ventricular dilatation and systolic dysfunction and accounts for up to 30–40% of heart failure. Genetic mutations have been identified in about 30% of patients with dilated cardiomyopathy. Mutations in the lamin A/C gene (*LMNA*) were reported to cause an autosomal dominant inherited form of dilated cardiomyopathy (hereafter referred to as *LMNA* cardiomyopathy). Profound metabolic deficits associated with dilated cardiomyopathy have already been reported^33^.

## Results

In order to obtain an aqueous solution of the HP metabolite suitable for *in vivo* studies, a systematic approach to improve the crucial steps of the PHIP-SAH procedure has been undertaken. This work has dealt with: (i) seeking for the best experimental solutions to enhance the polarization level at the ^13^C-carboxylate signal; (ii) obtaining a bio-compatible aqueous solution of the hyperpolarized metabolite.

**Figure 1:**
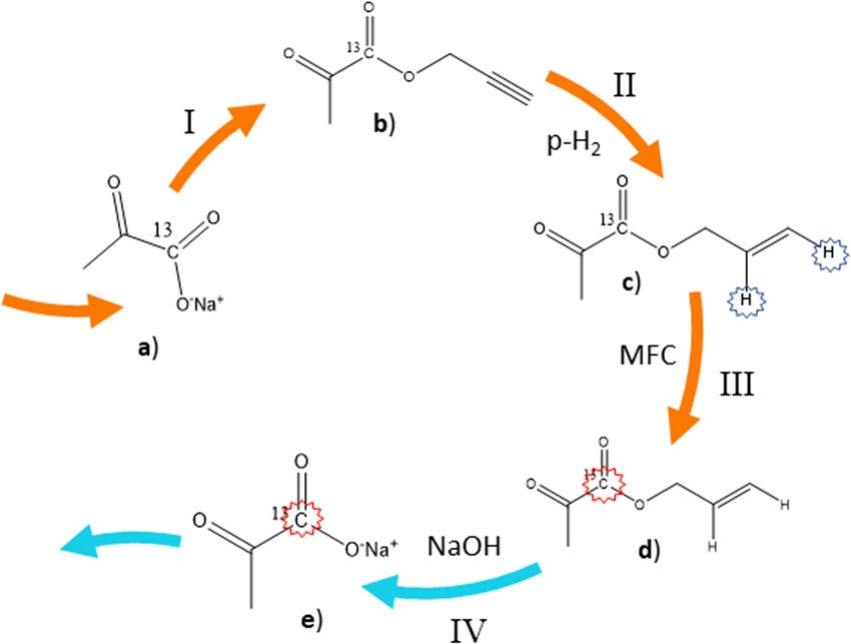
Schematic representation of the PHIP-SAH procedure. (I) functionalization of [1-^13^C]pyruvate with propargylic alcohol I (I) hydrogenation of the triple bond with parahydrogen; (III) polarization transfer from parahydrogen protons to the ^13^C carboxylate signal; (IV) hydrolysis of the ester with aqueous NaOH (0.1 M) and phase extraction in the aqueous phase. Orange arrows means that these passages take place in the organic phase; blue arrows: these reactions occur in the aqueous phase.

The polarization level observed on [1-^13^C]pyruvate, after hydrolysis of the ester precursor, depends on several factors, in particular on the efficiency of the parahydrogen singlet state addition to the substrate (hydrogenation, step II in Fig. 1), the polarization transfer step (carried out by means of MFC, step III in Fig. 1) and the maintenance of the ^13^C hyperpolarization during hydrolysis (step IV). Each step of the PHIP-SAH procedure has been investigated in detail in order to assess the most suitable experimental conditions. As reported by Cavallari *et al*., the application of controlled magnetic field cycle for spin order transfer from the parahydrogen protons to the ^13^C carboxylate spin, together with washing of the NMR tubes using a concentrated acidic hot solution, allowed to markedly increase the ^13^C hyperpolarization of the carboxylate signal of allyl-pyruvate (compound c in Fig. 1) and we observed 6.2 ± 0.3% ^13^C polarization on the ester.

Hydrolysis and extraction of sodium pyruvate in the aqueous phase (step IV, Fig. 1) have been carried out using a diluted aqueous base (NaOH 0.1 M), instead of the concentrated base (NaOH 1 M) reported in the proof-of-concept study^34^, in order to obtain a physiologically compatible solution of the HP product. The efficiency of the hydrolysis has been improved, as reported, thanks to the quick injection of the pressurized hot solution. Finally, the addition of the buffered acidic solution yielded the pH of the aqueous solution to the physiological values range (7–7.4).

Furthermore, the already reported addition of sodium ascorbate (0.05 M) to the base solution, limited significant the polarization loses during hydrolysis and the observed the ^13^C signal enhancement was 39,000 ± 5,600 with respect to thermal polarization, corresponding to 3.5 ± 0.5% polarization.

As far as the *in vivo* applicability of the product is concerned, an important change, with respect to the previously reported study, has been done in the organic solvents mixture used to carry out the hydrogenation reaction. In the previous work, activation of the catalyst precursor ([Rh(dppb)diene]^+^) in ethanol implied the use of a relatively high percentage of this hydrogenation co-solvent, that was subsequently extracted in the aqueous phase. In the herein reported experimental procedure, the catalyst activation step has been eliminated and a minimum amount of ethanol, about three equivalents with respect to the hydrogenation substrate, has been added to the catalyst dissolved in chloroform, in order to adjuvate the hydrogenation catalysis. This allowed to further improve the bio-compatibility of the final aqueous phase. Most importantly, the significant decrease of the amount of hydrogenation co-solvent has not caused any negative effect neither on the catalytic efficiency nor on the polarization yields. Moreover it has been observed that, while the catalyst activation implied the degradation of large part of the complex (see Figure S1), no significant degradation occurs in this case.

The concentration of [1-^13^C]pyruvate, after phase extraction, was determined to be 35 ± 5 mM. The relaxation time constant (T_1_) of the ^13^C carboxylate signal of pyruvate, measured on the 1 T MRI system used for the acquisition of the *in vivo* experiments, resulted to be 68 ± 0.5 s, in good agreement with previously reported studies^35^.

### Hyperpolarized [1-^13^C]pyruvate *in vivo* detection

**Figure 2:**
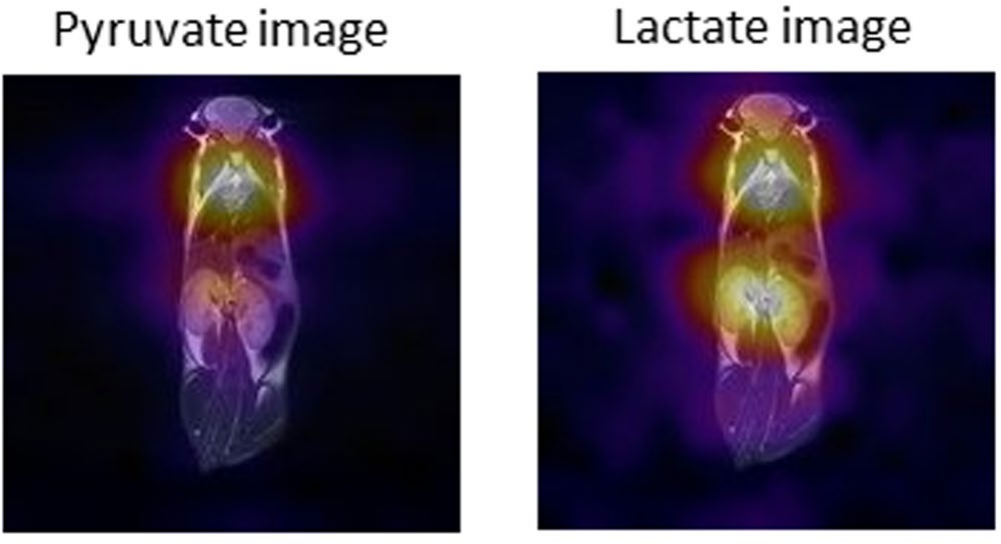
^13^C-Chemical Shift Imaging reporting of pyruvate and lactate distribution acquired on living mice, obtained upon the injection of a dose of HP [1-^13^C]-pyruvate. The spatial localization of each metabolite is shown upon overlapping the ^13^C-CSI results to the anatomical ^1^H-image (T_2_ weighted Fast Spin echo image). Each metabolite map is scaled individually and is displayed on a fire color scale so that the region of highest metabolite signal appears white and the lowest appears black.

^13^C-CSI (^13^C chemical shift imaging) acquisitions have been obtained within 15–20 s after the i.v. injection of a dose of HP [1-^13^C]pyruvate in living mice. Metabolite maps have been generated at the resonance frequencies of pyruvate and lactate and displayed as color overlays on the proton reference image corresponding to the center of the 20 mm ^13^C coronal slice (Fig. 2). Each metabolite map was scaled individually and displayed on a fire color scale. We found tissue-specific localization of the metabolites that was observed in all the acquired ^13^C-CSI studies, demonstrating that the most intense pyruvate signal is localized in the heart region whereas lactate formation is detected in both heart and kidneys.

**Figure 3:**
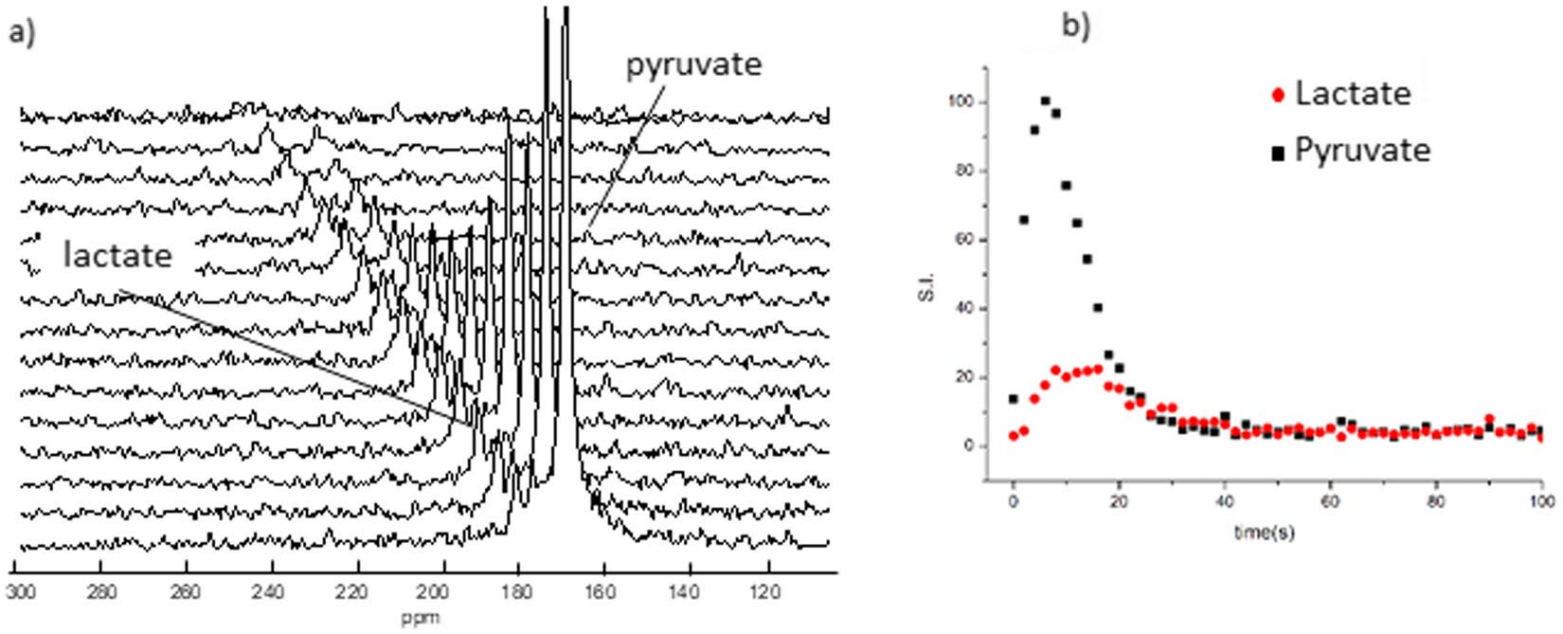
Dynamic metabolic study carried out on the heart of living mice. (**a**) stacked plot of ^13^C-MR spectra at 1T from a dynamic study on the heart of a WT mouse; spectra were acquired every 2 seconds from a 12 mm thick axial slice centered on the heart. The lactate peak is easily identifiable (182 ppm) ([1-^13^C]pyruvate signals 170 ppm). (**b**) dynamic curves of metabolite levels in the 12 mm slice centered on the heart, the metabolite level has been obtained from the integral of the metabolite peak.

Series of ^13^C-MR spectra acquired on a slab centered on the heart or the kidneys have shown the metabolic build-up of lactate (Fig. 3). Kinetic analysis of the metabolic exchange of the ^13^C label between [1-^13^C]pyruvate and endogenous lactate has been carried out by monitoring the time courses of the signals of both metabolites (Fig. 3).

The exchange rate of the ^13^C hyperpolarized label between [1-^13^C]-pyruvate and lactate has been obtained, in each experiment, using the model free approach based on the ratio of the total area under the curve (AUC) of the two metabolites^36^.

### ^13^C hyperpolarized pyruvate reports altered cardiac metabolism

**Figure 4:**
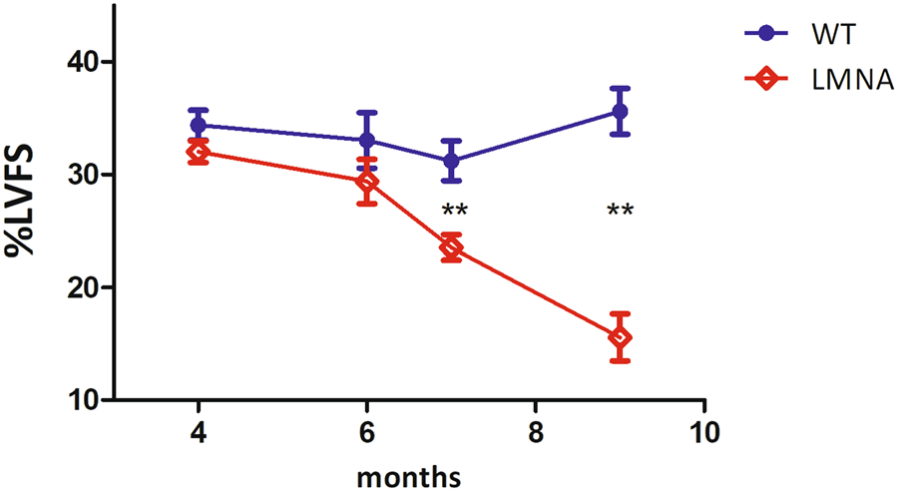
Left Ventricle Fractional Shortening obtained from echographic measurements carried out on WT and genetically modified *Lmna*^H222P/H222P^ mice. It can be clearly observed that LVFS in *Lmna*^H222P/H222P^ mice becomes lower (**P = 0.0065) than that in WT mice at 7 months of age and this difference is more significant at 9 months of age (**P = 0.0012).

**Figure 5:**
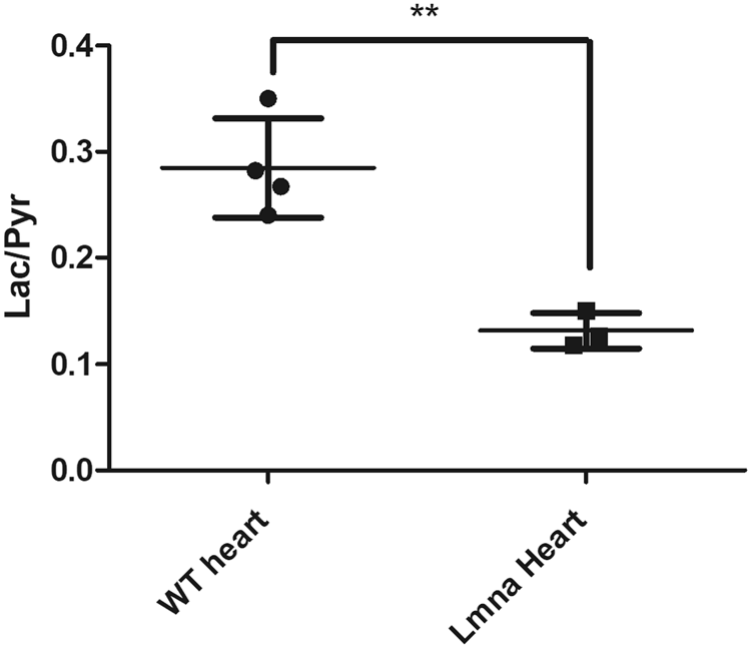
Lactate to pyruvate AUC ratios obtained from ^13^C-MR spectra acquired on a slice positioned on the cardiac region of a 6-months-old WT (dots) and a 6-months-old *Lmna*^H222P/H222P^ mouse (squares). The series of ^13^C-MR spectra were acquired following to the administration of HP [1-^13^C]-pyruvate.

A reprogrammed metabolic pattern in development of cardiomyopathy could be reflected in an abnormal ^13^C label exchange rate between the injected HP [1-^13^C]pyruvate and the endogenous lactate. We used *Lmna*^H222P/H222P^ mice, a model for dilated cardiomyopathy^32^. Male *Lmna*^H222P/H222P^ mice develop cardiac chamber dilation and decreased left ventricle fractional shortening detectable by echocardiography at 7 months of age (Fig. 4). In order to assess the reproducibility of the metabolic response following to the administration of the PHIP-SAH hyperpolarized substrate, a control wild-type and a *Lmna*^H222P/H222P^ mouse (6-months old) received four and three injections of HP [1-^13^C]pyruvate, respectively. The ^13^C HP label exchange rate between pyruvate and lactate was significantly lower in the heart of the *Lmna*^H222P/H222P^ mouse compared to the control one (Fig. 5).

**Figure 6:**
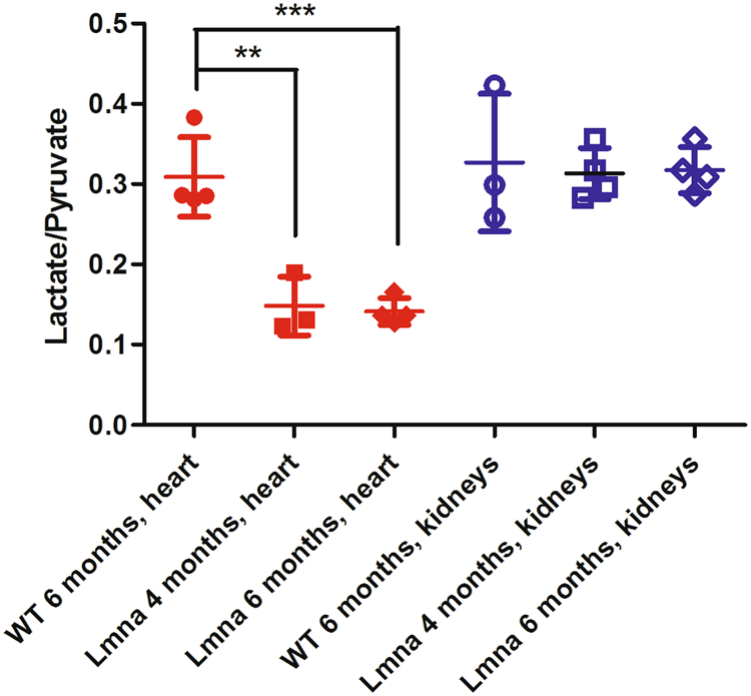
Lactate to pyruvate AUC ratios obtained from the ^13^C-MR spectra centered on the heart (filled red symbols) of mutant *Lmna* mice (4-months and 6-months old) and WT mice (6 months old). The lactate to pyruvate ratio in the heart of *Lmna*^H222P/H222P^ mice is significantly lower (**P = 0.005) than in WT mice, already at 4 months of age and becomes more significant (***P = 0.0007) on 6 months old *Lmna*^H222P/H222P^ mice. When the ^13^C-MR spectra are centered on the kidneys (open blue symbols) there is not any significant difference between mutant and WT mice.

The experiments have been repeated on 4-months old mice and the altered lactate/pyruvate ratio was already observed at this age in the heart of *Lmna*^H222P/H222P^ mice (Fig. 6), when cardiac function observed in the echocardiographic measurements was normal (Fig. 4).

Conversely, the lactate/pyruvate ratio obtained from the ^13^C MR spectra acquired on the slice centered on kidneys was the same in *Lmna*^H222P/H222P^ and control mice (Fig. 6). This finding support the view that the metabolic result obtained in the heart reports on differences in the metabolism of the cardiac tissue and not from impairment in the distribution of the HP metabolites in the blood pool.

## Discussion

This study shows that *in vivo* metabolic imaging investigations based on the administration of a dose of [1-^13^C]pyruvate obtained from the PHIP-SAH procedure is possible. ^13^C hyperpolarization has been markedly increased on pyruvate ester and on the hydrolysis product, with respect to the originally reported 2%. Magnetic field cycle has been optimized^37^ and 6.2 ± 0.3% hyperpolarization has been observed on the ^13^C carboxylate of allyl-pyruvate, while the remarkable value of about 10% has been back-calculated. The hydrolysis and phase extraction steps, carried out manually, took few tens of seconds (55–60 s). Considering the relaxation rate of the ^13^C carboxylate signal of [1-^13^C]pyruvate is 54 s, less than one half of the nascent polarization is retained on the ^13^C carboxylate signal (3.5 ± 0.5%). Nevertheless, this polarization appears already sufficient for carrying out *in vivo* studies, as shown recently^38^ using d-DNP hyperpolarized [1-^13^C]pyruvate. It is also worth noting that the herein reported results were obtained on a 1T-MRI scanner using a volume coil, thus showing that the ^13^C signals of pyruvate and lactate can be distinguished at this low magnetic field strength. Of course, higher magnetic field and more sensitive coils, such those applied in the study by Bastiaansen *et al*.^38^, in which 9.4T MRI scanner and ^13^C surface coil were used, would lead to better SNR and improved spectral resolution.

It follows that the hyperpolarization level will be markedly increased by adopting an automatized procedure with optimized technological solutions for each of the involved steps.

HP [1-^13^C]pyruvate has been used for assessing the *in vivo* metabolism of the heart tissue of genetically modified *Lmna*^H222P/H222P^ mice. Mutations in the *LMNA* gene induce several diseases, called laminopathies, the most important one is the cardiomyopathy^39^, i.e. a decrease of the cardiac contractile function, that is usually assessed by means of echocardiographic investigations. An alteration of cardiac mitochondrial metabolism has been demonstrated recently^40^. The herein reported results clearly show a marked decrease of pyruvate to lactate exchange kinetics in the heart muscle of *Lmna*^H222P/H222P^ mice. The reduced pyruvate/lactate exchange rate is a reporter of the general metabolic activity of the cells and might be due to either lower activity of the transporters (MCT) or altered cytosolic redox state. Pyruvate enters the cells through MCT transporters and, in the cytosol, the enzyme lactate dehydrogenase (LDH) catalyzes its interconversion to lactate, using NADH/NAD^+^ as coenzymes (Pyruvate + NADH ↔ Lactate + NAD^+^). As higher cytosolic oxidative potentials have already been observed in these mutant mice^40,41^, a reduced NADH/NAD^+^ ratio may lead to lower conversion of pyruvate into lactate in the heart of *Lmna*^H222P/H222P^ mice. The ^13^C-MRS acquisitions carried out on the kidneys support the view that the altered metabolism observed at the heart tissue is due to the *Lmna* mutations, while kidneys remain unaffected. Remarkably, the decreased ^13^C label exchange rate is clearly observed already at 4-months of age, when the decrease of the cardiac contractile function is not evident yet by echocardiographic investigations.

Here we report a technique to report cell metabolism alteration *in vivo*, in real time, in the affected tissue. We conclude that this methodology represents a noninvasive method of continuous and quantitative appreciation of cardiac metabolism *in vivo*, allowing longitudinal study of heart with altered metabolism, in real time. Consequently, one may expect that the easy access to HP [1-^13^C]pyruvate by PHIP-SAH methodology will prompt new comers to the use of this powerful metabolic imaging tool that was previously available only to the d-DNP equipped labs.

## Experimental Methods

### Hyperpolarization of [1-^13^C]Pyruvate

The PHIP-SAH procedure to obtain an aqueous solution of HP [1-^13^C]-pyruvate consists of the following steps: (i) synthesis of the ester from [1-^13^C]pyruvic acid and propargylic alcohol; (ii) hydrogenation of the unsaturated triple bond with parahydrogen in an organic solvent in the presence of a homogeneous catalyst; (iii) polarization transfer, from parahydrogen protons to the ^13^C carboxylate signal, by means of magnetic field cycle (MFC); (iv) hydrolysis of the ester by means of heated, pressurized aqueous base; (v) phase extraction of sodium [1-^13^C]-pyruvate retained in the aqueous phase; (vi) neutralization of the aqueous solution.

In the applied experimental work-up, the *para*hydrogenation of the propagyl ester of [1-^13^C]-pyruvate (2-propynyl-2-oxo-propan-[1-^13^C]oate) has been carried out using [Rh(COD)dppb]^+^ as catalyst in a chloroform/ethanol solution.

The catalyst has been dissolved in chloroform (100 μl), then the solution was frozen in liquid nitrogen, the hydrogenation substrate (3 μl) and three-four equivalents of ethanol were added.

The hydrogenation reaction was carried out in NMR tubes charged with the hydrogenation mixture (catalyst, substrate, solvents) and pressurized with parahydrogen (92% para-enriched hydrogen). Moreover, in the context of complete removal of paramagnetic impurities from the reaction environment, it has been found that extensive acidic washings of the glass tubes with concentrated HCl allowed to remove metal containing species that resulted in beneficial effects on the hyperpolarization level.

The hydrogenation reaction was carried out as follows: the NMR tube containing catalyst, substrate and solvent, pressurized with 92% para enriched hydrogen (6 bar), was heated at 353 K for 8 seconds, then vigorously shaken for 3 seconds and the reaction was allowed to continue for other 5 seconds before opening the NMR tube.

The polarization transfer from the parahydrogen protons to the ^13^C carboxylate signal was obtained by applying a magnetic field cycle (MFC). The NMR tube was placed in a mu-metal box (triple shield) containing a solenoid coil supplied with electric current for an exact control of the fast (diabatic) and slow (adiabatic) steps during the MFC. Magnetic field cycle has been optimized, through the accurate control of the intensities of the magnetic fields between which the sample is cycled as well as by finding the optimal speed for the passages to the low field (diabatic, fast) and back, to earth field (adiabatic, slow passage). The magnetic field was lowered from 1.5 μT to 50nT in 1 ms, then adiabatically increased to 10 μT in 4 seconds, using an exponential remagnetization profile. In order to measure ^13^C hyperpolarization on allyl-pyruvate, CDCl_3_ (300 μl) was added and the product solution was transferred into a syringe. The syringe was placed into the 1 T MRI scanner where a series of ^13^C small flip angle pulses (15° pulses) was applied^13^.C signal enhancement was measured with respect to a thermally polarized ^13^C enriched sample of sodium succinate -1,4-^13^C_2_, the observed S.E. resulted to be 70000 ± 3000 times with respect to thermal equilibrium at 1 T (6.3% polarization).

Hyperpolarization has been back calculated to be 10.3 ± 0.5 at time zero, considering that i) the relaxation time constant (T_1_) of this ^13^C carboxylate at earth’s magnetic field is 89 seconds and ii) the time delay between end of MFC and the acquisition of the first ^13^C-MR spectrum is 45–50 seconds.

Hydrolysis of the ester and phase extraction in the aqueous phase was achieved using 260 μl of an aqueous solution of NaOH 0.1 M and sodium ascorbate (0.05 M), heated (350 K) and pressurized with an inert gas, using an home-made device, in order to obtain quick injection of the aqueous base with the organic phase. Following mixing of the heated base solution with the organic phase, a suspension of small droplets of the organic phase into the aqueous one has been instantaneously formed and hydrolysis of the ester occurred in few seconds (6–8 s) at the interface between the two phases.

Sodium ascorbate, a scavenger of radicals, allowed to maintain the ^13^C hyperpolarization of the carboxylate moiety during hydrolysis of the ester and extraction of sodium pyruvate in the aqueous phase^37^.

An acidic buffer (100 ul HEPES 100 mM, pH 5.3) was then added to obtain physiological pH. The aqueous solution containing the HP metabolite was collected in a syringe, then transferred *via* a plastic vial to another syringe for the *in vivo* injection. Transfer of the aqueous solution through the vial was meant to remove traces of organic solvent from the aqueous solution. It has been determined that CDCl_3_ contaminates the aqueous phase at a 0.014% level (<1 mg/L). At the end, 240 ± 20ul of hyperpolarized [1-^13^C] pyruvate aqueous solution were collected for the i.v. injection. The residual Rh concentration in the injected solution has been measured by ICP analysis and was 0.03 mM 30*10^−9^ mol/ml. Considering that 200 ul were injected in each mouse, the average weight of which was 27 ± 2 g, the amount of injected Rh(I) corresponds to 0.22 *10^−6^ mol/Kg, that is about three orders of magnitude lower than the reported LD50 values for various Rh(I) complexes in mice (Rh(I)carbonyl acetylacetonate, LD50 mouse i.p. 0.78*10^−3^ mol/kg; RhAcetate LD50 Mouse i.p. 1.75*10^−3^ mol/Kg)^42,43^.

^13^C polarization has been measured, on the 1 T MRI system used for *in vivo* experiments, on a syringe containing the aqueous solution of [1-^13^C]-pyruvate obtained from phase extraction whose ^13^C-MR spectrum was acquired using a small flip angle pulse at 1 T, as reported above. The polarization decay rate of the ^13^C carboxylate has also been measured on the 1 T MRI scanner applying a series of small flip angle pulses (15° pulses). The relaxation time constant has been obtained from fitting of the experimental data with the pulse-corrected exponential decay function ${M}_{oss}={M}_{0}\cdot \,\cos \,{(\theta )}^{(n-1)}\cdot exp(\,-\,n\cdot {\rm{\Delta }}t/{T}_{1})$ (where n is the number of the pulse and *θ* is the pulse angle).

^13^C hyperpolarization level of the ^13^C carboxylate of sodium [1-^13^C]pyruvate was back-calculated at time zero, i.e. at the end of MFC, just before the reaction with the aqueous base, considering that the T_1_ of the ^13^C carboxylate is 54 s and the time delay between hydrolysis and the acquisition of the first ^13^C MR spectrum is 50–60 s. This yielded the value of 9.7 ± 1.5%, i.e. the same as that estimated above for the ester precursor. The concentration of [1-^13^C]-pyruvate has been measured acquiring a thermally polarized ^13^C NMR spectrum (14 T, Bruker Avance NMR system) of a portion of the aqueous phase.

### *In vivo* experiments

In this study, 4-months-old (n = 3) and 6-months-old (n = 4) male *Lmna*^H222P/H222P^ mice and 6-months-old male WT mice (n = 4) were investigated. During MRS/MRI acquisitions, mice were anesthetized, with 3% Isoflurane in 1.5 l/min oxygen. A catheter was introduced through the tail vein for the intravenous administration of the solution of hyperpolarized pyruvate before the mouse was transferred to the MR scanner. While the mouse was in the scanner, anesthesia was maintained using a continuous delivery of isoflurane (1–1.5%) and breathing was monitored using a plastic balloon placed under the animal. Injection of the aqueous solution of hyperpolarized [1-^13^C]-pyruvate (0.25 mmol/Kg) was carried out in a single bolus and lasted 5–6 seconds. At the end of the experiment, mice were placed on a heated pad and awakened. The animals completely recovered from the experiments and did not show any apparent change of the vital signs (breath and heart rate) associated to the injection of the HP product. The animals received a maximum of four injections of the HP product solutions.

This study complied with the guidelines for use and care of laboratory animals and was approved by the Italian Ministry of Health (General Direction of Animal Health and Veterinary Drugs) (n. 1012/2015-PR) and by the University of Torino Ethical Board Committe.

### MRS-MRI experiments

The MR scanner used for this study was a 2 M Aspect Imaging scanner operating at 1 T, equipped with a dual tuned ^1^H/^13^C volume transmit-receive radiofrequency coil.

For spatial localization, ^13^C dynamic studies were performed using a modified ^13^C-CSI sequence, without phase encoding (^13^C-CSI_noPE sequence, Aspect Imaging) that allowed to acquire space-selective ^13^C-MR spectra. Spectra from an axial slab spanning 12 mm (slice centered either on the heart or on the kidneys) were recorded every 2 seconds using a 15° flip angle. Acquisition of the space-selective series of MR spectra (32 spectra, 256 spectral points, acquisition bandwidth 3000 Hz, repetition time 2 seconds) was started at the beginning of the injection of the aqueous solution of hyperpolarized pyruvate (that took 5–6 seconds).

^13^C-CSI images were also acquired in four *Lmna* mice. A low-flip angle CSI acquisition (^13^C_CSI Aspect Imaging) was used with 256 spectral points, an acquisition bandwidth of 3000 Hz and a repetition time of 64 ms. An excitation pulse with 10° flip angle was used to excite a 20 mm coronal slice centered on the mouse body, with a 80 × 80 mm FOV and a phase encoding matrix 10 × 10. CSI were acquired over a 4 seconds period starting 15–17 seconds after the onset of pyruvate injection.

High resolution T_2_-weighted anatomical images (coronal images) were acquired to provide the anatomical basis for spatial localization of the ^13^C hyperpolarized signals. A fast spin echo (FSE) sequence was used and coronal images were obtained with FOV 80 × 80 mm, matrix size 160 × 160 and slice thickness 1.5 mm.

### Echocardiographic measurements

In order to assess the heart functionality, transthoracic echocardiography was performed in the mice using a Vevo 770 (Visualsonics, Inc., Canada) device equipped with a 30 MHz probe. Mice were slightly anesthetized with 0.5–1% isoflurane in O2. An LV M-mode tracing was obtained using the 2D parasternal short-axis imaging as a guide. LV end-diastolic (LVEDD) and LV end-systolic (LVESD) diameters were calculated from the mean of at least three separate cardiac cycles. Fractional shortening was calculated as [(LVEDD − LVESD/LVEDD) *100].

### Data processing

The FIDs obtained from the ^13^C dynamic studies were zero-filled to 512 points and Fourier transformed. The obtained ^13^C-MR spectra were interpolated, using a Matlab function, with three Lorenzian curves corresponding to the ^13^C signals of pyruvate, hydrated pyruvate and lactate. The area of each peak was obtained from the integral of each peak.

The kinetic analysis of the exchange rate of the ^13^C label between pyruvate and lactate was carried out using the model free approach based on the ratio between the total area under the curve (AUC) of the product and the injected metabolite^36^. When this model is used, the time courses of integrals from hyperpolarized lactate and pyruvate is summed and the ratios of the total lactate/total pyruvate is taken.

^13^C-CSI data were zero filled to 512 points in the time domain and 20 × 20 points in the k-space domain prior to Fourier transform. The data point corresponding to the frequency of maximum signal intensity for each metabolite (pyruvate and lactate) was chosen to generate frequency-selective metabolite images. The metabolite maps were presented as color overlays on a grayscale proton reference image.

## Electronic supplementary material

Supplementary Information
