# Supplementary material for: The 13C hyperpolarized pyruvate generated by ParaHydrogen detects the response of the heart to altered metabolism in real time
Source: Sci Rep. 2018 May 30;8:8366. doi: 10.1038/s41598-018-26583-2 (PMC5976640; doi:10.1038/s41598-018-26583-2)
Supplement: Supplementary file 1 — Supplementary Information [file 41598_2018_26583_MOESM1_ESM.docx]

Supplementary material to

**The ^13^C hyperpolarized pyruvate generated by ParaHydrogen detects the response of the heart to altered metabolism in real time**

*Eleonora Cavallari,* Carla Carrera,* Matteo Sorge,* Gisèle Bonne,^§^ Antoine Muchir, ^§^ Silvio Aime,* Francesca Reineri**

**Dept. of Molecular Biotechnologies and Health Sciences, University of Torino, Torino (Italy)*

*^§^* *Sorbonne Université, Inserm UMRS974, Center of Research in Myology, Institut de Myologie, G.H. Pitie-Salpetriere, Paris, France*

Hydrogenation Catalyst

In order to characterize the hydrogenation catalyst ([RhCODdppb]^+^, see structure in figure S1) , ^31^P-NMR spectra have been acquired before and after the activation, i.e. hydrogenation of the coordinated diene. When the complex is dissolved in chloroform, before activation, the doublet due to direct scalar coupling ^31^P-^103^Rh demonstrate that all the ligand is coordinated to the metal. After hydrogenation, COD is replaced by the solvent (structure II figure S1). The ^31^P NMR shows the formation of a large amount of free phosphine (singlets due to free phosphine and phosphine oxide) while only a percentage (about 20%) of the total amount is still bounded to the metal. This implies the formation of free Rh(I) that dismutates into Rh (metal) and Rh(II) (paramagnetic).


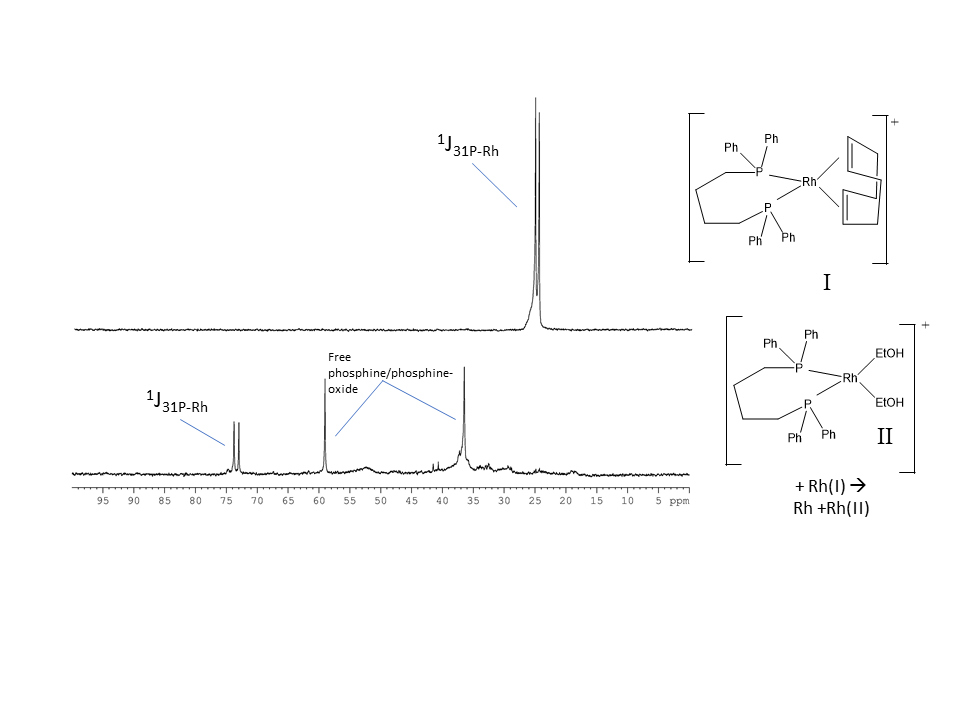


**Figure S1.** ^31^P-NMR spectra of a) the catalyst [(RhCODdppb)]^+^ dissolved in chloroform, the doublet is given by ^1^J_P-Rh_ coupling; b) the catalyst after activation in ethanol: a large percentage of free phosphine can be observed (singlets), while only about 20% of the ligand is bound to the metal.
